# Long noncoding RNAs in development and cancer: potential biomarkers and therapeutic targets

**DOI:** 10.1186/s40591-015-0042-6

**Published:** 2015-06-12

**Authors:** Roshan Fatima, Vijay Suresh Akhade, Debosree Pal, Satyanarayana MR Rao

**Affiliations:** Molecular Biology and Genetics Unit, Jawaharlal Nehru Center for Advanced Scientific Research, Jakkur, Bangalore 560064 India

**Keywords:** LncRNA, Development, Cancer, Biomarker, Therapy

## Abstract

Long noncoding RNAs are emerging as key players in various fundamental biological processes. We highlight the varied molecular mechanisms by which lncRNAs modulate gene expression in diverse cellular contexts and their role in early mammalian development in this review. Furthermore, it is being increasingly recognized that altered expression of lncRNAs is specifically associated with tumorigenesis, tumor progression and metastasis. We discuss various lncRNAs implicated in different cancer types with a focus on their clinical applications as potential biomarkers and therapeutic targets in the pathology of diverse cancers.

## Introduction

The ‘Central Dogma’ of life describes the flow of genetic information from DNA to proteins involving RNA as an intermediate. The view of DNA being the store house of genetic information and proteins as its functional manifestation dominated the field of biology for several decades. However, the proposal of the famous RNA world hypothesis marked the beginning of an era where RNA was attributed more recognition in terms of cellular and physiological functionality rather than being considered only as a messenger between DNA and proteins [1]. After the completion of Human Genome Project it became evident that only a small portion of the genome encodes proteins [2, 3]. Further, advancements in tiling array and high throughput analyses revealed that the mammalian genome is pervasively transcribed [4, 5] and it was speculated that the large number of noncoding RNAs may reflect transcriptional noise. However, recent developments in the field of RNA biology have consolidated the fact that noncoding RNAs (ncRNAs) are indeed crucial molecules playing diverse regulatory roles in development and disease. On the basis of their main biological functions, ncRNAs are broadly classified as structural and regulatory ncRNAs. Structural ncRNAs have been known since a long time because of their role as essential components of the protein synthesis machinery [6]. These include transfer RNA (tRNA), ribosomal RNA (rRNA), small nuclear RNA (snRNA) and small nucleolar RNA (snoRNA). Regulatory ncRNAs include small interfering RNA (siRNA), microRNA (miRNA), piwi-RNA (piRNA) and long noncoding RNA (lncRNA) [7–9]. LncRNAs are arbitrarily defined as non coding transcripts of more than 200 nt in length. Most lncRNAs annotated till date have been reported to lack protein coding capacity, albeit few of them have the capacity to code for small peptides that had not been identified previously [10]. Based on their genomic location, lncRNAs are classified as sense lncRNA (lncRNA sequence overlapping with the sense strand of a protein coding gene), antisense lncRNA (lncRNA sequence overlapping with the antisense strand of protein coding gene), bidirectional (lncRNA sequence located on the opposite strand of a protein coding gene), intronic (lncRNA derived from an intron of a gene) and intergenic (lncRNA derived from region between two genes). In the present review we focus on the general features of lncRNA, their mechanisms of action and their role in development and cancer.

### Similarities and differences between lncRNA and mRNA

Irrespective of the major differences between mRNA and lncRNA with reference to their protein coding capacity, they share some common features as well. Similar to mRNA, most lncRNAs are transcribed by RNAPolII machinery and actively transcribed lncRNA genes possess histone modification signatures similar to that of protein coding genes [11, 12]. The ‘K4-K36 domain’ that refers to the distinctive chromatin signature of H3K4me3 modification at the promoters and H3K36me3 modification along the gene body of RNAPolII transcribed genes is present on a host of non-protein coding multi-exonic transcripts [11]. Furthermore, majority of lncRNAs are also polyadenylated and the pathway of biogenesis of lncRNA and mRNA cannot be distinguished from each other [13]. Studies have also revealed similarities between lncRNA and the 3’UTR region of mRNA mainly with respect to their secondary structures, sequence composition and thermodynamic parameters [14, 15]. Sequence conservation is a feature that distinguishes lncRNA and protein coding RNA. Many studies including recent lncRNA datasets identified from different species have shown the poor conservation of lncRNA sequences across species as compared to protein coding genes [16–18]. However, within their sequence, many lncRNAs have regions which exhibit very high conservation suggesting that key functional domains may be the ones that retain their identity over the evolutionary time period.

### Regulation of gene expression by lncRNAs

**Fig. 1:**
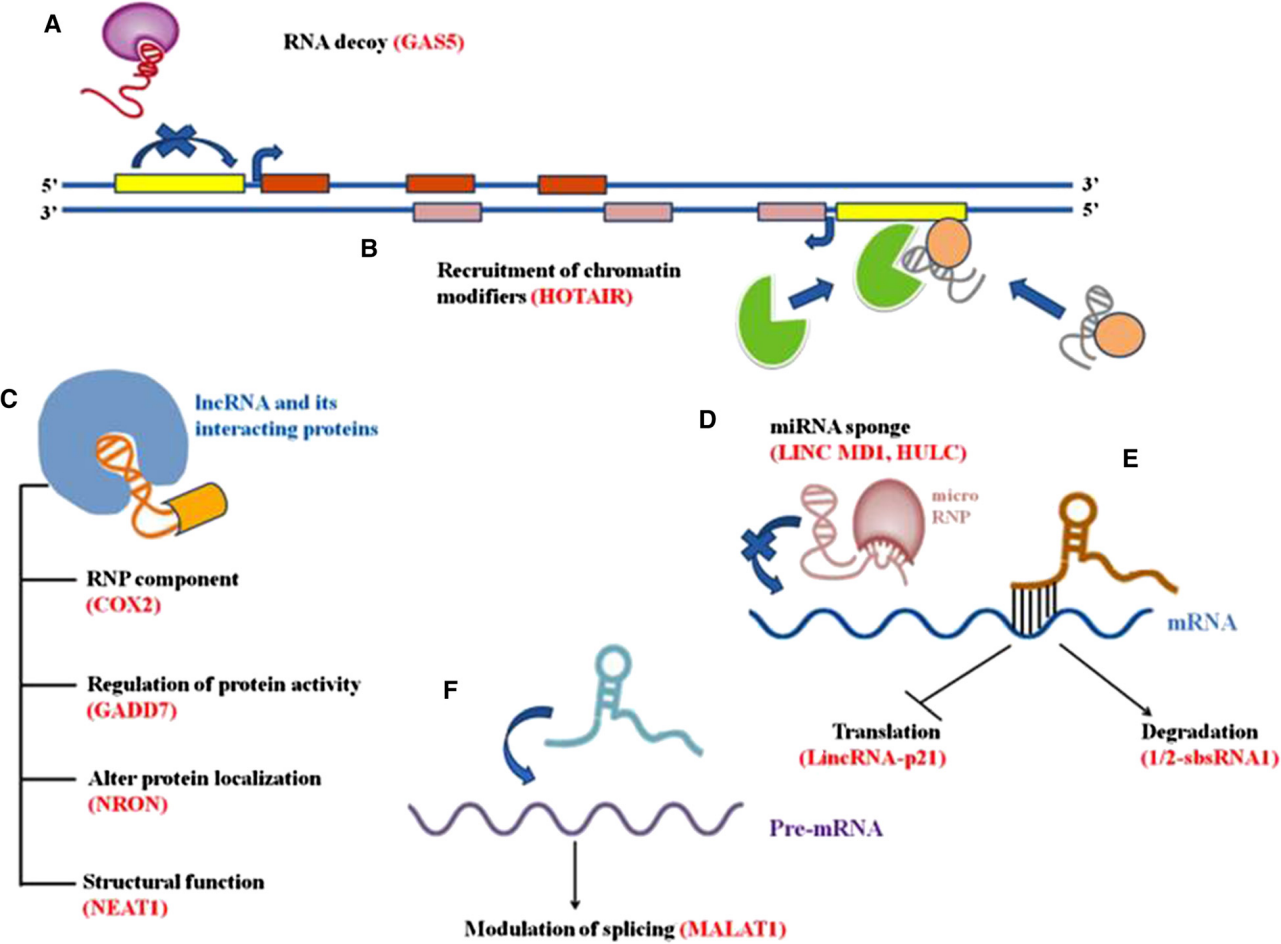
Diverse mechanisms of lncRNA function. Various studies have elucidated different mechanisms of function by lncRNA. One example of lncRNA for each mechanism is mentioned in the bracket. **a**) LncRNAs can function as decoys by binding to a transcription factor and preventing its action on the target DNA. **b**) LncRNAs modulate gene expression by recruiting chromatin modifiers. **c**) LncRNAs regulate various biological processes by being a part of RNP component, regulating the activity or localization of a particular protein and playing a structural role in organization within the nucleus. **d**) LncRNAs act as miRNA sponges by titrating the miRNAs away from their mRNA targets. **e**) LncRNAs modulate the translation and degradation of their mRNA targets. **f**) LncRNA can modulate the splicing of pre-mRNA. lncRNA, long non coding RNA; mRNA, messenger RNA; RNP, Ribonucleoprotein

It is being increasingly recognized that lncRNAs play a critical role in modulating genetic networks and signal transduction pathways during development and their deregulation leads to disease phenotypes [19, 20]. Several molecular mechanisms have been delineated for lncRNA mediated regulation of gene expression [21]. These molecular mechanisms include a) LncRNAs acting as decoys by binding to transcription factors and preventing the binding of these factors to their regulatory DNA elements [22]; b) Formation of triple helix with target DNA sequences [23]; c) LncRNAs titrating out miRNAs from their regulatory mRNA targets by binding to the specific miRNAs (miRNA sponge mechanism) [24]; d) LncRNAs as scaffold, which is one of the most common mechanisms employed by diverse lncRNAs [25, 26], e) LncRNAs acting as tethers to recruit protein partners resulting in the formation of functional ribonucleoprotein complexes [27]; f) Modulation of mRNA translation [28]; g) Modulation of splicing [29] and h) mRNA degradation [30]. Further, lncRNAs can serve as precursors for small RNAs like piRNAs, miRNAs or snoRNAs which can further perform their regulatory functions [31–33]. Other than their regulatory role in gene expression, lncRNAs also contribute to the organization of different nuclear structures [34, 35]. These mechanisms are pictorially depicted in Fig. 1. Besides, lncRNAs broadly regulate gene expression at epigenetic, transcriptional, post transcriptional levels and by cell-cell signaling through hormones as discussed below.

### Epigenetic mode of gene regulation by lncRNAs

A large number of lncRNAs remain in the nucleus and play an essential role in shaping the epigenome either by genomic imprinting or through chromatin remodeling as described below.

#### By genomic imprinting

Genomic Imprinting refers to the phenomenon of epigenetic silencing of an allele inherited from either of the parents [36]. Short stretches of DNA known as Imprinting Control Regions (IRCs) play a critical role in imprinting of multiple genes [37]. Interestingly, it has been observed that the imprinted regions show significant association with ncRNAs, which mediate the silencing by diverse mechanisms like chromatin remodeling and enhancer competition [38].

#### Through chromatin modifying complexes

The principal means by which most of the lncRNAs regulate gene expression is by recruiting chromatin remodelers to facilitate histone modifications at specific gene loci either for the repression or activation of the target genes [39]. Various lncRNAs have been shown to employ chromatin modifying complexes like Polycomb Repressive Complexes 1 and 2 (PRC1 and PRC2) [40–43], CoREST (CoRepressor for element-1-silencing transcription factor) [44], SMCX (Smcy homolog, X-linked), [45], G9a [46], LSD1 (Lysine Specific Demethylase 1) [47], Trithorax (Trx) activating complex [48], etc. to regulate gene expression, as discussed in detail later in this review.

### Transcriptional regulation of gene expression by lncRNAs

Recent studies have elucidated the fact that several lncRNAs modulate gene expression by specifically associating either at the promoters or the enhancers of their target genes.

#### Promoter-associated lncRNAs (plncRNAs/ pRNAs)

Divergent transcription at the promoter regions of various genes gives rise to lncRNAs which in turn regulate the transcription of the neighboring genes [49–52]. For example, Hung et al. [53] studied the chromatin landscape around transcription start site (TSS) of 56 cell cycle associated genes and showed that 49 of these genes are associated with at least one lncRNA. One of these, PANDA (Promoter of CDKN1A Antisense DNA Damage Activated RNA), produced from the CDKN1A promoter region, shows p53 dependent induction after DNA damage and aids in cell proliferation by inhibiting the apoptotic genes [53]. Further, Negishi et al. [54] reported a novel lncRNA, APTR (Alu-mediated p21Transcriptional Regulator) that represses the transcription of CDK inhibitor p21 by recruiting PRC2 complex to the p21 promoter. LincRNA-p21 is another promoter associated lncRNA that acts as a p53 dependent tumor suppressor. It gets localized to promoters of genes that are repressed by p53, facilitating their inhibition through hnRNP-K [55, 56].

#### Enhancer-associated lncRNAs (elncRNAs/ eRNAs)

Enhancers are critical regulatory elements required for the tight developmental and tissue specific regulation of gene expression and hence it is not surprising that the number of enhancers in mammalian genome far exceeds the number of protein coding genes [57, 58]. Very recently, several studies have revealed that enhancers give rise to lncRNAs that are suggested to be crucial for mediating gene regulation, both positively and negatively, by mediating chromosomal looping [59–63]. Additionally, super enhancers have been reported which consist of clusters of enhancers, mainly associated with genes involved in maintenance of cell identity [64, 65]. Super enhancers are also known to be associated with lncRNAs. For example the lncRNA, CCAT-1 L (Colorectal Cancer Associated Transcript 1-Long isoform) transcribed from an upstream super enhancer locus of the oncogene, Myc (Myelocytomatosis) functions as an eRNA and plays a role in transcription regulation of Myc [66].

### Post transcriptional regulation

LncRNAs are also widely implicated in the post transcriptional regulation of mRNAs including splicing, transport, translation and degradation. For example, MALAT1 is involved in splicing events [67], discussed in detail later. Certain other lncRNAs have been implicated in stabilizing and promoting the translation of mRNAs by extended base pairing with them [68]. In addition, lncRNAs can also facilitate the inhibition of mRNA translation or decay by partial base pairing with the 3’UTR sequences through their Alu elelemts in Staufen-mediated manner [30].

### Regulation through hormone responsive genes

Some lncRNAs regulate gene expression through their interaction with hormone receptors. For example, SRA (Steroid receptor RNA Activator) lncRNA is a coactivator of various steroid hormone receptors like GR (Glucocorticoid Receptor), AR (Androgen Receptor), ER (Estrogen Receptor) and PR (Progesterone Receptor) [69]. GAS5 is another lncRNA that participates in hormone mediated gene regulation [22].

### LncRNAs in early mammalian development

Widespread studies have established that lncRNAs participate in a variety of mammalian developmental processes like regulating lineage commitment and cell fate decisions, in organogenesis, in imprinting of alleles during early development and also in specification of the body pattern. A majority of the lncRNAs exhibit a tissue-specific expression pattern [12], which helps in fine tuning and coordinating the context-dependent signals to regulate the cellular physiology when compared to the more ubiquitously expressed protein molecules. Interestingly, many of the lncRNAs involved in regulating development contribute to various disease pathologies including cancer, when altered [70, 71]. Recent studies have identified cancer stem cells as the main players that drive cancer progression in most cases. Cancer stem cells bear striking similarities with the on setters of development i.e., embryonic stem cells. Both these type of cells possess unlimited proliferative capacity and harbor the potential to migrate to specific destinations by undergoing epithelial to mesenchymal transition. Under such circumstances, it would be worthwhile to study how the multitude of lncRNAs that govern developmental cues can also lead to various disorders, developmental in nature or otherwise.

#### Dosage compensation

An excellent example of genome level regulation has been provided by the discovery of XIST (X inactive-specific transcript). Being involved in inactivating one of the pair of X chromosomes, its expression is restricted mainly to females and further only from the X-chromosome that will be inactivated in the future [72]. Analysis of the conservation pattern between mouse and human XIST reveals identical stretches of sequences with interspersed non-conserved regions, suggesting that over evolution, principle functional domains have been retained. The locus has been shown to transcribe a couple of lncRNAs including REPA and TSIX. While REPA is derived from XIST and acts to recruit the PRC2 complex to inactivate the future X_i_ (inactive X chromosome) via H3K27 trimethylation of the chromatin [73], TSIX is the antisense repressor of XIST and prevents inactivation of the future X_a_ (active X-chromosome) [74]_._ Like the negative regulator of XIST, there exists a positive regulator, JPX, that in turn is produced from the X-inactivation center and exerts its action in *trans* to activate XIST on X_i_ [75]. This TSIX-JPX switch for X_a_-X_i_ provides a wonderful illustration of RNA-based transcriptional control.

Loss of function studies for XIST further emphasize its importance in mammalian development. Marahrens et al. [76] generated a targeted partial deletion of the *Xist* gene and interestingly discovered that mutant males were unaffected by the deletion along with mutant females who inherited the deleted gene maternally. However, mutant females containing the paternally inherited deletion showed death early during embryogenesis. This was attributed to the expression of both X chromosomes in the extra-embryonic tissues that led to abnormalities in the development of the embryo proper.

#### Patterning of the body axes

The specification of the anterior-posterior body axis and determination of the positional identity of individual cells as well as organs is governed by a group of homeodomain containing proteins, encoded by the *Hox* clusters of genes. LncRNAs have been associated with this phenomenon, a predominant one being HOTAIR [77]. It represents a classical example of the *trans* mode of action of lncRNAs as it is expressed from the *HoxC* locus in mammals but exerts its action at the *HoxD* locus. HOTAIR recruits the PRC2 complex at the target locus resulting in spreading of H3K27 trimethylation over the region and additionally interacts with the LSD1/REST/co-REST complex to perform lysine 4 demethylation, exemplifying the functioning of lncRNAs as molecular ‘scaffolds’ [25]. The *Hox* locus is in fact quite a storehouse of lncRNAs. HOTTIP is expressed at the 5’ end of the *HoxA* locus and recruits the WDR5/MLL complex across the locus by chromosomal looping, bringing about H3K4 trimethylation and subsequent gene transcription. Interestingly, its strength of action on the Hox genes decreases with increasing distance from its own site of transcription [78]. While HOTTIP has a more distal pattern of expression, another lncRNA at the *HoxC* locus, FRIGIDAIR has a function in anterior patterning [21]. The complex interplay between proteins and lncRNAs at such gene loci at the *Hox* loci is thus crucial in proper embryonic development.

Targeted deletion at the *Hotair* locus has revealed that the lncRNA is as essential as the HOX proteins for the proper development of the embryo [79]. Its absence leads to malformation of the skeletal system, massive derepression at several loci including that of *HoxD* and certain imprinted loci like *Dlk1*, *Igf2* (paternally imprinted) and *H19*, *Meg3* (maternally imprinted) amongst others. Perturbations in these genes further alters gene expression pattern *in vivo* leading to abnormalities during development.

#### Genomic imprinting

LncRNAs have also been implicated in genomic imprinting of specific alleles, a phenomenon that is a part of the early developmental regime. AIR (Antisense Igf2r RNA) is expressed in an antisense direction from the Igf2r (Insulin-like growth factor type2 receptor) locus, is maternally imprinted and assists in the imprinting of certain paternal genes like *Slc22a2* and *Slc22a3*, expressed upstream from *Air* [80]. Early during embryonic development, in the placenta, AIR acts at the Slc22a3 promoter but not at the Igf2r promoter, by interacting with H3K9 methyl transferase, G9a [81]. KCNQ1OT1 is another example of a lncRNA participating in allelic imprinting. Being maternally imprinted and paternally expressed antisense to the Kcnq1 locus, it is involved in gene repression at various loci in the paternal genome that have been classified as ubiquitously imprinted (*Kcnq1*, *Cdkn1c*, *Phlda2* and *Slc22a18)* or placental-specific imprinted *(Osbpl5*, *Tssc4* and *Ascl2)* [82]. The lncRNA interacts with both G9a and PRC2 components to bring about imprinting as early as 3.5 to 5.5 dpc of embryonic development thereby playing an important role in specifying parental-specific gene expression [83].

LncRNA H19 is also involved in allelic imprinting, being expressed from the *Igf2* locus and itself being paternally imprinted [84, 85]. It is highly expressed from the maternal allele during the blastocyst stage and later in endodermal and mesodermal tissues, but is restricted in expression only to skeletal tissues in the adults [84]. Knockout of the *H19* gene results in mutant animals that are viable and fertile, showing an overweight phenotype probably due to a gain of biallelic expression of the previously imprinted Igf2 locus [85]. At the IGN (Imprinted Gene Network) locus, H19 acts to repress several genes including *Igf2*, *Slc38a4* and *Peg-1* by interacting with the methyl-CpG-binding domain protein, MBD1 [86]. The recruitment of this mediator protein to the IGN loci directs imprinting by bringing additional histone methyltransferases that drive repression of gene expression. Further, H19 acts as a precursor for the microRNA miR-675 that regulates placental growth [86].

LncRNAs have been well characterized in many cellular contexts and shown to help in maintenance of pluripotency of stem cells, in adult progenitor cell proliferation as well as in the differentiation of specific tissues [87–93]. Furthermore, their involvement in early mammalian development and in human diseases like cancer underlines their importance as an integral component of the pathways that regulate diverse physiological processes [94]. In view of this, the role of lncRNAs in cancer would be dealt with in detail in the upcoming section with special focus on their clinical and therapeutic relevance.

### LncRNAs in cancer

Cancer arises due to accumulation of genetic and epigenetic alterations in cells. Gain or loss of chromosomes has also been frequently observed in cancer cells. Several signal transduction pathways like Wnt/ β Catenin, MAPK, TGFβ, p14ARF/p53, PI3K/ AKT etc. are altered in the malignant cells which seem to produce their own growth factors and attain replicative immortality, enhanced angiogenesis and proliferation. Further, they evade growth repressors, escape apoptosis and acquire the ability of metastasis and invasion [95]. Transcriptome profiling of tumor cells has elucidated a central role for the vast noncoding landscape of the human genome in tumorigenesis. Specifically, long noncoding RNAs are emerging as key players in genetics and pathogenesis of cancer and their dysfunction is closely associated with cancer development, progression and metastasis; reviewed in [96–104]. While some lncRNAs are oncogenic by nature and drive cancer conditions when up-regulated, some others act as tumor suppressors and cause cancer only when they are down-regulated [105]. Some of the important lncRNAs deregulated in cancer, their mechanism of action and their potential clinical applications are discussed below.

H19 is among the earliest lncRNAs discovered and identified to be key factor regulating gene expression [106]. Expression of H19 is developmentally orchestrated and in turn it determines the repression of multiple genes through genomic imprinting [107, 108]. Interestingly, this lncRNA itself is produced from a paternally imprinted, maternally expressed gene at 11p15.5 locus, adjacent to the oppositely imprinted IGF2 (Insulin like Growth Factor2) gene. It produces a 2.3 kb spliced, capped and polyadenylated lncRNA conserved between rodents and human and also is processed to an miRNA, miRNA-675 [109]. A recent study by Monnier et al. [86] has shown that H19 silences the genes in the Imprinted Gene Network (IGN) through MBD1 (Methyl CpG-binding domain protein1), which is responsible for the repressive histone mark H3K9me3. Though the knockout of H19 is not lethal in mice [110], its over-expression, either due to loss of imprinting (LOI) at H19 locus or due to the loss of tumor suppressor gene p53 [111], or under the influence of the oncogene, Myc [112], leads to the activation of genes involved in angiogenesis, cell survival and proliferation [113, 114], triggering several malignancies like liver [115, 116], breast [117], colorectal [118], esophageal [119], lung [120], pancreatic [121], gastric [122], bladder [123] and cervical [124] carcinomas suggesting an oncogenic function for this RNA. In fact level of H19 expression shows significant correlation with tumor grade and is a potential biomarker for various cancers [114, 123, 125, 126]. In contrast, miR-675, the miRNA derived from H19, exhibits antagonistic behavior and functions as a tumor suppressor by repressing the IGF1R (Insulin like Growth Factor 1 Receptor) expression [127], thus the levels of these two transcripts help in maintaining cellular homeostasis.

KCNQ1OT1 (KCNQ1 Overlapping Transcript 1) is another imprinted, paternally expressed 91.5 kb transcript produced from the KCNQ1 locus, a few hundred kilobases away from H19 [128]. It regulates gene expression epigenetically by interacting with chromatin remodeling complexes like PRC1, PRC2 and G9a to bring about silencing of the KCNQ1 locus [129–131]. It is a cis regulatory RNA, the aberration of which is associated with Beckwith-Wiedemann syndrome (a congenital overgrowth syndrome) [132, 133], colorectal cancer [129], hepatocellular carcinoma [134] and pediatric adrenocortical tumors [135].

ANRIL (Antisense Noncoding RNA at INK4 Locus), also known as p15AS, is an antisense transcript of CDKN2B gene at the 9p21.3 locus. It has several alternatively spliced isoforms including 3.9 kb and 34.8 kb transcripts [26, 136, 137]. Misexpression of ANRIL is associated with a variety of diseases including cancer [138–140]. ANRIL brings about changes in gene expression by epigenetic means as it binds to both PRC1 and PRC2 and mediates gene silencing at the INK4b-ARF-INK4a locus [26]. It specifically associates with SUZ12, (Suppressor of Zeste 12 homolog), a subunit of PRC2, and mediates the repression of p15, a tumor suppressor gene [26], and consequently inhibition of ANRIL induces p15, resulting in reduced cell proliferation.

XIST (X-inactive-specific transcript, ~17 kb), one the earliest lncRNAs to be discovered [27], is expressed mainly in female somatic cells. It is transcribed from the Xic (X inactivation Center) on the X chromosome and spreads along and coats the chromosome from which it is transcribed in order to epigenetically silence it in cis by recruiting PRC2 [72, 141–144], thus achieving dosage compensation in males. Deregulation of XIST leads to loss or gain of X chromosomes resulting in a variety of female, male and non sex specific cancers [145–147], demonstrating the participation of lncRNAs in maintaining genomic stability. In female cancers like breast, ovarian and cervical cancers, the inactive X chromosome (Xi or the Barr body) is conspicuously absent in the malignant cells, while its duplication was also observed in some cells [148], due to XIST deregulation. Further, a majority of female cancer cell lines exhibited multiple copies of the active X chromosome (Xa), which is acquired either due to duplication of Xa or due to reactivation of Xi [147]. In fact the aberrant expression of XIST results not only in aberrant ploidy of X chromosomes but also in the increased resistance of cancer cells to chemotherapy [145].

Interestingly, XIST is expressed in males also, specifically in the transcriptionally inactive XY body in spermatocytes [149], though it does not seem to be required for the inactivation of XY body since male mice lacking XIST undergo normal spermatogenesis and silencing of X linked genes [150]. Notably, Xist is known to be over-expressed in Testicular Germ Cell Tumors (TGCTs) and also in patients with Klinefelter’s syndrome (47XXY). In both these cases, super numerical X chromosomes were observed which is suggested to contribute to oncogenesis [151, 152]. Moreover, XIST RNA is detectable in the plasma of such patients and has emerged as a serum biomarker for both these disease conditions [153, 154]. X chromosomal duplications were also frequent in normal XY men with male breast cancer [155, 156].

Not only in male and female cancers, XIST is implicated in sex independent cancers as well, mainly in lymphomas and leukemias. Expression of XIST is lost is these cancers resulting in extra active X chromosomes in both male and female patients of non-Hodgkin lymphoma [157–159]. Thus, lncRNAs not only play an essential role in the regulation of individual genes but they also control the copy number of chromosomes as well.

HOTAIR (HOX Transcript Antisense Intergenic RNA) is a 2.2 kb lncRNA produced from the HOXC gene cluster on chromosome 12 (12q13.13) and is involved in the *trans* silencing of genes at HOXD locus on chromosome 2 [25, 77]. It provides a typical example of lncRNA regulation of gene expression through the chromatin remodelers. It serves as a scaffold to anchor multi-protein complexes and has a remarkable ability of binding to distinct chromatin repressors. Specifically, its 5’ end binds to PRC2 while its 3’ end binds to LSD1 (Lysine Specific Demethylase 1A), which in turn interacts with CoREST (Co-Repressor for Elements-1-Silencing Transcription factor) and REST (Repressor for Elements-1- Silencing Transcription factor), setting off long term epigenetic silencing of target chromatin region through H3K27Me3 mark [25, 77].

HOTAIR is known to repress several tumor and metastasis suppressor genes like HOXD10 (Homeobox D10), PGR (Progesterone Receptor), PCDH10 (Protocadherin10), PCDHB5 (Protocadherin Beta 5), JAM2 (Junctional Adhesion Molecule 2), etc. [160–162] and therefore its up-regulation leads to a variety of malignancies like primary/ metastatic breast cancers [161, 163–165], hepatocellular [166–168], colorectal [162], gastrointestinal [169, 170] and non-small cell lung carcinomas [171]. It is an oncogenic lncRNA associated with cell proliferation, invasiveness and reduced apoptosis and thus serves as a diagnostic and prognostic marker for multiple cancers.

While the above discussed lncRNAs are involved in gene regulation at epigenetic level, certain other lncRNAs are involved in transcriptional/ post transcriptional events, as exemplified by NEAT1 and MALAT1, the aberrant expression of which results in cancer.

NEAT1 (Nuclear Enriched Abundant Transcript 1) gene produces two transcripts, the 3.7 kb NEAT-1-1 short isoform and 23 kb NEAT-1-2 long isoform. NEAT1 is widely expressed across several tissues, though the expression of long isoform is much lower as compared to the short isoform. NEAT1 localizes to the paraspeckles in the nucleus [172, 173] and plays a crucial role in transcriptional and post-transcription regulation of gene expression and its knockdown leads to disintegration of paraspeckles [34]. In fact NEAT1 and NEAT2 (MALAT1) exhibit transcription dependent binding on human genome over hundreds of active genes. NEAT1 is induced strongly in breast cancer cells and is also involved in the transformation of myeloid cells into acute promyelocytic leukemia (APL) [174]. Further, it is highly upregulated in ATRA (All Trans Retinoic Acid) induced differentiation of NB4 (APL) cells which could be inhibited by specific siRNA for NEAT1 [174]. Silencing of NEAT1 in Burkitts lymphoma cells results in reduced viability, increased apoptosis and abnormal morphology suggesting its oncogenic nature [175].

MALAT1 (Metastasis Associated Lung Adenocarcinoma Transcript1) is another prominent lncRNA implicated in a variety of cancers. Also known as NEAT2 (Nuclear Enriched Abundant Transcript 2), it is a 7.5 kb RNA transcribed from the 11q13.1 locus, expressed broadly across various normal human tissues [67, 176]. MALAT1 undergoes post-transcriptional processing to yield a short t-RNA like cytoplasmic mascRNA (malat1 associated small cytoplasmic RNA) and a long MALAT1 transcript that localizes to nuclear speckles and is involved in splicing events. It specifically localizes to the nuclear speckles of SR (Serine Arginine) proteins, which are required for both constitutive and alternative splicing and the levels of MALAT1 directly influence the level of phosphorylated SR proteins [29, 67]. It is over-expressed in a variety of cancers like lung [176, 177], liver [178, 179], bladder [180, 181], pancreatic [182], cervical [183], breast [184], prostate [185], colorectal [186] and uterus [187]. It is specifically linked to high metastasis rate and poor prognosis in non-small cell lung cancer patients [188]. Further, its overexpression is shown to bring about cell survival, proliferation, migration and promotion of epithelial-mesenchymal transition by activating Wnt signaling *in vitro* in urothelial carcinoma [180, 181] and hence it is suggested to be involved in cell motility. Notably, while the over expression of MALAT1 is associated with severe consequences, its knockdown in mice is neither lethal nor shown to cause any defects *in vivo* [189].

Certain lncRNAs like SRA and GAS5 mediate gene regulation through interaction with hormone receptors and lead to cancer when deregulated, as discussed below.

SRA (Steroid receptor RNA Activator), an lnc RNA transcribed from the 5q31.3 locus, 2 kb in size, is a coactivator of various steroid hormone receptors as discussed earlier. It has been reported that the SRA1 gene plays a dual role and codes both for a protein (SRAP) and an lncRNA (SRA), by alternative splicing [190–192]. The levels of this protein and the lncRNA are suggested to impact tumorigenesis and tumor progression by varying the expression of target genes [98]. SRA is a part of RNA-protein (RNP) complex and brings about the *trans* activation of genes through its interaction with the AF1 domain of nuclear receptors [69]. Its over-expression and consequent deregulated hormone signaling is associated with breast [193, 194], uterine, ovarian [195] and prostate [190] cancers.

GAS5 (Growth Arrest Specific 5) gene at 1q25.1 locus produces two splice variant lncRNAs and its introns also give rise to several snoRNAs [196]. GAS5 functions as a tumor suppressor and facilitates normal growth arrest and apoptosis through repression of GR mediated transcription [22, 197]. It specifically interacts with DNA binding domain of GR and inhibits the binding of GR to its target genes including cIAP2 (cellular Inhhibitor of Apoptosis 2), bringing about apoptosis, independent of other stimuli in cancer cells. Moreover, GAS5 has also been suggested to repress progesterone receptor and androgen receptor in a ligand dependent fashion [22, 196]. It also mediates the inhibition of mTOR (mammalian Target of Rapamycin), which regulates protein synthesis, cell growth and proliferation. This fact is corroborated by the observation that anti proliferative effect induced by Rapamycin could be repressed by silencing of GAS5 in primary T cells as well as in leukemic cells [198]. In turn, GAS5 is regulated by a negative feedback loop with miR-21 [199]. Down-regulation of GAS5 and/or its snoRNAs along with genetic aberrations at its locus were found to be associated with poor prognosis in several cancers like breast cancer [197, 200], prostate cancer [201], leukemia [198], gastric cancer [202], cervical cancer [203], renal cell and Bladder cancer [204, 205].

#### Telomere associated lncRNA, TERRA in human cancers

Telomeres, the ends of chromosomes, are composed of a hexanucleotide repeat, TTAGGG in vertebrates which protects and prevents end to end fusion in chromosomes [206]. The telomere repeats shorten after each cell cycle in normal cells which can lead to chromosome instability and cell death [207]. Most of the cancer cells overcome this adversity by Telomerase activity which requires ncRNAs. The telomerase enzyme has a protein component called TERT (Telomerase Reverse Transcriptase) and an RNA component called TERC (Telomerase RNA Component) [208]. Apart from TERC, a group of lncRNA transcripts named TERRA (Telomeric Repeat containing RNA) derived from the subtelomeric loci has recently been identified. TERRA localizes to and brings about the hetrochromatin formation in telomeres, a conserved phenomenon in eukaryotic cells [209, 210]. TERRA is suggested to be a negative regulator of telomerase and leads to cancer when down-regulated [211, 212].

#### T-UCRs in human cancers

Transcribed Ultraconserved Regions (T-UCRs) are evolutionary highly conserved sequences between orthologous regions of the human, rat and mouse genomes [213, 214]. They give rise to transcripts of 200–779 nt in length that show tissue specific expression. Many of the T-UCRs show altered expression in cancers like chronic lymphocytic leukemia [214], colorectal carcinoma [214], neuroblastomas [215, 216], hepatocellular carcinoma [217] and prostate cancer [218]. The T-UCRs can be targeted by miRNAs and offer opportunities for novel therapeutic interventions [214, 219].

While the above mentioned lncRNAs are implicated in multiple cancers, certain other lncRNAs have so far been linked only to specific cancer types so far as discussed below.

HULC (Highly Up-regulated in Liver Cancer), 1.6 kb in size, is transcribed from the 6p23.3 locus. It was discovered by Panzitt et al. [220] with the help of Hepato Cellular Carcinoma (HCC) specific microarray as the most highly up-regulated lncRNA in this cancer. Like a typical mRNA, it has two exons and a poly A tail and strongly localizes to the cytoplasm and co-purifies with ribosomes but does not code for any protein. It sequesters miRNAs and is involved in the inhibition of miRNA mediated repression. Liu et al. [221] reported that the SNP, rs7763881 in HULC locus was significantly associated with HCC susceptibility in HBV (Hepatitis B Virus) carriers. Further, knockdown of CREB (cAMP response element-binding protein) expression as well as use of a PKA (Protein kinase A) inhibitor resulted in down regulation of HULC, revealing that phospho CREB is required for activation of HULC [222]. HULC is oncogenic in nature and highly up-regulated in both tumors and plasma of HCC patients but it is not detected in any other tissues or their cancers [220]. Thus it serves as a specific non-invasive biomarker for HCC [223]. Moreover, it is not expressed in primary colorectal cancers but is detected in colorectal cancers that metastasize to liver showing its specificity for the hepatic tissue [224].

HEIH (High Expression In HCC) is a 1.6 kb oncogenic polyadenylated transcript generated from the 5q34.3 locus. Yang et al. [225] examined the differentially expressed lncRNAs between HBV related HCC and normal tissues and one of the RNAs, HEIH, was studied in detail. HEIH was shown to play a critical role during cell cycle and associated with EZH2 (Enhancer of Zeste Homolog 2), a critical component of PRC2 and represses the EZH2 target genes [225]. The levels of HEIH were found to be significantly associated with HCC recurrence and post-operative survival of patients and thus it serves as an independent prognostic factor [225].

PCA3 (Prostate Cancer Antigen 3; also known as DD3, Differential Display Code3) is derived from the 9q21.22 locus, and transcribed as three alternately spliced isoforms of 0.6 kb, 2 kb and 4 kb [226]. It is expressed at low levels in normal prostate and highly up-regulated in >95 % of prostate tumors, but not in any other normal or cancer tissue. It is a potent biomarker detectable in urine of prostate cancer patients with higher specificity and sensitivity as compared to PSA (Prostate Specific Antigen) [226, 227].

PCGEM1 (Prostate Cancer Gene Expression Marker), 1.6 kb in size, derived from the 2q32 locus, it is one of the earliest oncogenic lncRNAs discovered. It is regulated by Androgen Receptor (AR), a transcription factor which has a critical role in the prostate gland development [228]. PCGEM1 is highly elevated in prostate cancer, especially in patients with a family history of prostate cancer. It promotes cell growth, proliferation and inhibits doxorubicin induced apoptosis. Overexpression of PCGEM inhibits PARP cleavage and delays the induction of p53 and p21 resulting in increased chemo-resistance. It plays an important role during carcinogenesis and serves as a specific biomarker for prostate cancer [229].

PCAT1 (Prostate Cancer Associated ncRNA Transcript 1) is a 7.8 kb lncRNA transcribed from the 8q24.13 locus, up-regulated in both metastatic and high grade localized prostate tumors. Prensner et al. [230] identified 121 prostate cancer associated transcripts (PCATs) by RNA sequencing analysis of prostate cancer tissues of which PCAT1 is the most highly up-regulated. Knock down of PCAT1 in androgen dependent prostate cancer cell line resulted in alteration of hundreds of genes [230]. It has also been reported that PCAT1 has an important role to play in double strand break repair and inhibits homologous recombination [231]. It is a transcriptional repressor of DNA repair genes like BRCA2 tumor suppressor and in turn is regulated by PRC2. Overexpression of PCAT1 is linked to increased sensitivity to PARP inhibitors due to decrease in RAD51 foci formation [231]. PCAT1 is a negative prognostic marker for prostate cancer [230].

These prostate specific lncRNAs are proving to be very useful in the clinic as diagnostic and prognostic markers in Prostate cancer since the traditional markers like PSA have only limited prognostic value [232].

Anti-NOS2A (Anti Nitric Oxide Synthase 2A) is a 1.9 kb intronless polyadenylated lncRNA expressed in meningomas and glioblastomas, transcribed from the NOS2A (17q23.2) locus. It is involved in the negative regulation of NOS2A, which plays an important role in the neuronal differentiation [233].

HOTAIRM1 is a 483 bp transcript generated from the HOXA cluster. It is a regulator of hematopoiesis and its down-regulation results in the inhibition of several HOXA genes required for hematopoiesis. In APL (Acute Promyelocytic Leukemia), differentiation of hematopoietic precursors gets blocked at promyelocytic stage due to chromosomal translocations involving Retinoic Acid Receptor alpha (RAR α) gene. ATRA (All Trans Retinoic Acid) is used to treat this condition and HOTAIRM1 was found to be induced in ATRA mediated differentiation of APL cells [234], showing that its down regulation is linked to the disease phenotype.

DLEU1 and DLEU2 (Deleted in lymphocytic Leukemia 1 and 2) are two lncRNAs produced from the 13q14.3 tumor suppressor locus [235] which is deleted in lymphomas and hematopoitic cancers like Chronic Lymphocytic Leukemia (CLL) [236, 237]. DLEU1 and DLEU2 are regulated epigenetically and in turn regulate a cluster of genes that influence NF-kB expression. Interestingly, expression of the protein coding genes at the 13q14.3 locus is altered but they are not associated with any SNPs, whereas the promoter regions of the two lncRNAs exhibit demethylation/activation marks in CLL suggesting that the lncRNAs regulate the protein coding genes in cis. Further, DLEU2 splice variants are the precursors of cell cycle inhibitory miRNAs, miR-15a and miR-16-1, which are suggested to be involved in CLL [238, 239].

Apart from the individual lncRNAs associated with cancer, several genome wide microarray analyses in recent years have shed light upon hundreds or thousands of lncRNAs that are deregulated in various cancers [240–244], further corroborating the fact that lncRNAs are important players involved in the development and progression of cancers.

### LncRNAs as potential biomarkers and therapeutic targets

LncRNAs are not only providing us with a new perspective to our understanding of disease mechanisms but also furnishing fresh therapeutic opportunities [245–247]. In fact lncRNAs have an advantage over protein coding genes in that their expression is more tissue specific, thus making them attractive as biomarkers and therapeutic targets. LncRNAs are remarkably stable in body fluids and tissues, proving to be valuable biomarkers in liquid biopsies, facilitating the avoidance of invasive procedures [105, 248, 249]. Their distribution and levels can be evaluated with the help of various techniques like *in situ* hybridization, qPCR, transcriptome profiling etc. [248], which can be used to assess the disease progression and/or recovery with a particular treatment regimen.

LncRNAs can be targeted therapeutically by a variety of approaches including RNAi mediated gene silencing, antisense oligonucleotides, plasmid based targeting, through small molecule inhibitors and by gene therapy as discussed below (Reviewed in 105, [250–252].

#### RNAi mediated down regulation of specific lncRNAs for therapy

RNA interference mediated silencing of genes involved in various diseases provides a direct approach to selectively inhibit target molecules. This can be achieved through different agents like siRNA (small interfering RNAs), shRNAs (short hairpin RNAs), and miRNAs. Even though most of the lncRNAs are known to show nuclear localization, various studies have revealed that they can still be targeted by RNAi mediated intervention [39].

The lncRNA HOTAIR is upregulated and serves as a diagnostic and prognostic biomarker for breast, liver, gastro-intestinal, lung and colorectal carcinomas [161–171, 253]. Down regulation of HOTAIR expression by siRNA is associated with reduced viability and invasiveness and induction of apoptosis in breast, hepatocellular and pancreatic cancers [167, 254, 255]. Furthermore, knockdown of HOTAIR also enhanced the sensitivity of cancer cells to tumor necrosis factor alpha based immune response and also to chemotherapeutic agents like cisplatin and doxorubicin [167]. The lncRNA PCA3 is highly up-regulated in prostate cancer and is a potent biomarker detectable in urine [226]. siRNA mediated down-regulation of PCA3 significantly inhibited growth and viability of prostate cancer cells and also reduced the expression of AR target genes [256], suggesting it can be a potential therapeutic target. LncRNAs PCAT1, PRNCR1, PCGEM, PlncRNA1 and PCAT18 are also highly up-regulated in aggressive prostate tumors and have been suggested as biomarkers and therapeutic targets for the same [257, 258]. siRNA/shRNA based silencing of these lncRNAs in prostate cancer cell lines inhibited cell proliferation and induced apoptosis by decrease in AR expression [258, 259]. The lncRNAs H19, HULC, HEIH, MVIH are highly upregulated in hepatocellular cancer and are valuable biomarkers for the same [116, 220, 225, 260]. siRNA/shRNA mediated silencing of these transcripts resulted in altered expression of several genes and reduced growth of tumors in xenografts indicating they are potential therapeutic targets [220, 225]. The lncRNAs H19, UCA1, CUDR, HIF1A-AS are reliable biomarkers and potential therapeutic targets for bladder cancer [123, 261–265]. MALAT1 is a prognostic marker for lung, gastrointestinal and several other cancers [176–188]. shRNA mediated silencing of MALAT1 inhibited the migration and invasive potential of adenocarcinoma cells and cervical cancer cells respectively [177, 178]. Down-regulation of MALAT1 by siRNA in HEPG2 cell line results in reduction in tumor progression, cell motility and viability along with induction of apoptosis [179]. The lncRNA CCAT2 is up-regulated in colorectal cancer and can be targeted by specific miRNAs [Table 1].

**Table 1:** List of lncRNAs associated with different cancers

LncRNA	Size	Locus	Mechanism	Nature	Related cancer/ Biomarker/Therapeutic target	References
ANRIL	3.9 kb, Multiple isoforms	9q21.3	Regulates CDKN2A/2B locus by recruiting PRC1/PRC2	Oncogenic	Prostate cancer, Leukemia, other diseases	[26, 137–140]
anti-NOS2A	1.9 kb	17q23.2	Down regulates Nos2A	Oncogenic	Meningomas and Glioblastomas	[233]
lncRNA-ATB	2.4 kb	14q11.2	Activated by TGF-β	Oncogenic	Hepatocellular carcinoma	[310]
BC200	0.2 kb	2p21	Translational modulator	Oncogenic	Multiple cancers	[283]
CCAT1	2.6 kb, 5.2 kb	8q.24	Regulates Myc by long range chromatin loops	Oncogenic	Colorectal, gall bladder cancer	[66, 316, 317]
CCAT2	0.34 kb	8q24.21	Involved in Microsatellite stability	Oncogenic	Colorectal, lung, breast cancers	[305, 306, 318]
CRNDE	10.3 kb, Multiple transcripts	16q12.2	Interacts with PRC2, CoREST, regulated by Insulin, IGF	Oncogenic	Colorectal cancer, glioma	[284, 285]
CUDR	2.2 kb	19p13.12	Involved in drug resistance	Oncogenic	Lung, cervical, colon and Bladder cancer	[261, 262]
H19	2.3 kb	11p15.5	Imprinting	Oncogenic	Liver, esophagal, breast, bladder, Pancreatic, colorectal, gastric, cervical	[84–86, 106–127]
HEIH	1.6 kb	5q34.3	Represses PRC2 target genes through EZH2	Oncogenic	Hepatocellular carcinoma	[225]
HOTAIR	2.2 kb	12q13.13	Chromatin modification by binding to PRC2, LSD1	Oncogeneic	Breast, liver, lung, gastrointestinal and colorectal	[160–171]
HULC	0.5 kb	6p24.3	Interactor of CREB	Oncogenic	Hepatocellular carcinoma	[220–224]
KCNQ1OT1	91.5 kb	11p15.5	Imprinted lncRNA, binds to PRC2 & G9a	Oncogenic	Colorectal cancer	[82, 83, 128–135]
MALAT1	~8 kb	11q13.1	Modulates alternative splicing	Oncogenic	Colorectal and breast cancers	[29, 67, 176–189]
NEAT1	3.7 kb, 23 kb	11q13.1	Transcriptional and Post transcriptional regulation	Oncogenic	Prostate cancer and leukemias	[34, 174, 175]
ncRAN/ SNHG16	2 kb	17q25.1	Interacts with N-myc	Oncogenic	Bladder, colorectal cancer, neuroblastoma	[286, 287]
PCA3	0.6, 2 kb, 4 kb and 23 kb	9q21.2	AR signaling	Oncogenic	Prostae Cancer	[226, 227, 257]
PCAT1	7.8 kb	8q24.21	Inhibits homologous recombination	Oncogenic	Prostate cancer	[230, 231]
PCGEM	27 kb	2q32	Activates AR regulated genes	Oncogenic	Prostate cancer	[228, 229]
PCNA-AS1	384 bp	20p12.3	Increases stability of PCNA mRNA	oncogenic	Hepatocellular carcinoma	[311]
PlncRNA-1	24.5	21q22.12	Interacts with AR	Oncogenic	Prostate cancer	[259]
PRNCR1	13 kb	8q24.22	Activates AR regulated genes	Oncogenic	Prostate cancer	[288]
PVT1	>300 kb, Mutiple transcripts	8q24	Interacts with p53	Oncogenic	Liver, breast, ovarian, colorectal, gastric, nonsmall cell lung cancer and leukemia	[289–295]
SChLAP1/ PCAT114	224.8 kb	2q31.3	Inhibits binding of SWI/SNF on genome	Oncogenic	Prostate cancer	[296]
SRA	2 kb	5q31.3	Regulation through steroid hormones and PRC2	Oncogenic	Prostate, breast, ovarian and uterine cancers	[69, 98, 190–195]
TUG1	6.7 kb, splice variants	22q12.2	Interacts with PRC2 to repress target genes, induced by p53	Oncogenic	Urothelial and non small cell lung cancer	[297, 298]
UCA1	1.4 kb, 2.2 kb, 2.7 kb	19p13.12	Regulates cell cycle through CREB	Oncogenic	Bladder and Breast cancer	[312–315]
AK126698	3.8 kb	1q24.2.	Cisplatin resistance through Wnt signaling	Tumor Suppressor	Non-small cell lung cancer	[299]
BANCR	693 bp	9q21.11	Regulates MAPK pathway	Tumor suppressor	Melanoma, retinoblastoma, lung	[307–309]
GAS5/ SNHG2	Multiple lnc and snoRNAs	1q25.1	Hormonal regulation (GR)	Tumor Suppressor	Breast, prostste, Gastric, cervical and renal cell cancers	[22, 196–205]
LET	2.6 kb	15q24.1	Repression by HDAC3 under hypoxia conditions	Tumor Suppressor	Lung, liver and colorectal cancer	[300]
LincRNA-p21	3 kb	6p21.2/ NA?	p53 dependent repression of genes through hnRNP-K	Tumor suppressor	Lymphoma, lung, colorectal carcinomas	[28, 55, 56, 280]
MEG3	1.6 kb, splicing isoforms	14q32.2	Positive regulator of p53	Tumor suppressor	Prostate, bladder, Pituitory adenocarcinomas, meningoma	[301–304]
PTEN-P1 (Pseudogene)	~4 kb	9p13.3	Enhances PTEN expression	Tumor suppressor	Prostate, colon cancers	[278]
XIST	19 kb	Xq13.2	Imprinting, binds to PRC2	Tumor suppressor	Breast, ovarian and cervical cancers	[72–76, 141–159]

Although RNAi based therapeutic agents are used to target lncRNAs in cell lines quite effortlessly, *in vivo*, they would require suitable delivery vehicles like liposomes, nanoparticles or viruses for proper cellular uptake, prevention of their degradation or accumulation in liver. Nevertheless, several RNAi based therapies are in clinical trials [266, 267], though there is still a need for further improvements for safe and effective remedies.

#### Antisense Oligonucleotides (ASO) mediated therapy

Antisense oligonucleotides are short (13–25) single stranded DNA oligonucleotides complementary to RNA of interest. ASOs are modified to avoid degradation by nucleases and in turn they induce RNase H mediated cleavage of their target transcripts. Several ASOs, mainly those targeting mRNAs are already in advanced clinical trials [268, 269], while two ASO based drugs to treat Cytomegalovirus retinitis and high blood cholesterol have already been approved by FDA [270, 271]. Similar approaches are being developed to target cancer related lncRNAS. Accordingly, AntagoNATs, ASOs that target antisense lncRNA, are being employed to up-regulate specific mRNAs/ proteins by silencing the corresponding antisense lncRNA [272]. AntagoNATs are modified not only in their 5’ and 3’ termini but also in their backbone in order to make them more stable and to enhance their cellular uptake. Thus ASOs have an advantage over siRNAs which are usually unstable and hard to be targeted into tumor cells *in vivo* [273]. Notably, ASO (Antisense Oligonucleotide) mediated knockdown of MALAT1 inhibited the metastasis in human lung cancer cells in a mouse xenograft model [188]. Despite the promise, poor cellular uptake and cytotoxicity remain as matters of concern for ASOs.

#### Small molecule inhibitor mediated modulation of lncRNAs

The molecular interactions of lncRNAs with their interacting protein partners can be blocked by small molecule inhibitors that mask the binding sites on their interactors [251]. Accordingly, the interaction of HOTAIR with PRC2 or LSD1 can be inhibited with the help of small molecular inhibitors to reduce the metastasis in breast cancer [274]. Alternately, in another approach, small molecule inhibitors or specific oligonucleotides can be designed to bind and change the secondary structure of lncRNAs and thus inhibit their interaction with binding partners [251, 275]. Targeting the lncRNA-protein interactions would not only lend tissue and developmental specificity but also has an advantage over targeting only RNAs or proteins since lncRNAS mediate regulation of gene expression essentially through their protein partners. Furthermore, this method is also superior to RNAi based methods which may suffer from off target effects. Moreover small molecules are easier to be administered and exhibit a better cellular uptake than ASOs, siRNAs or viral vectors. However, this approach needs a better understanding of RNA-protein interactions and identification of additional molecules that target RNA.

#### Plasmid based therapy

In a novel therapeutic approach, a plasmid, BC-819/ DTAH19, has been developed which carries a diphtheria toxin subunit under the regulation of H19 promoter. When this plasmid is injected into the tumor, it brings about the reduction in tumor size due to the production of high level of diphtheria toxin in human trials of bladder cancer [276]. This method attempts to reduce the tumor size in general rather than targeting any specific lncRNA and has shown encouraging results in recent times in other cancers like lung, colon, pancreatic and ovarian cancer as well [101].

#### Gene therapy

Some lncRNAs are down-regulated in tumor samples as compared to normal tissues. The lncRNA PTCSC3 (Papillary Thyroid Carcinoma Susceptibility Candidate 3) is down-regulated in thyroid tumors [277]. PTENP1, a pseudogene of PTEN, is down-regulated in colon carcinoma [278]. MEG3 is downregulated in meningioma and glioma [279]. LincRNA-p21 is down-regulated in lymphoma, lung carcinoma [56] and colorectal cancer [280]. Delivery of beneficial tumor suppressor RNAs can be attempted with the help of gene therapy in such cases [247, 251].

In summary, though the above discussed means of targeting long noncoding RNAs for cancer therapy looks very promising in cell lines, the delivery of therapeutic agents to their specific targets in actual patients *in vivo* would be quite challenging and effective strategies need to be developed for the same [281]. Although, trials on mouse models have shown some hope, but many of the lncRNAs are primate/ human specific and cannot be investigated *in vivo* in knockdown/ knockout models in mice. Another point of concern is the fact that even though it has been well established that altered expression of lncRNAs is associated with various cancers, it has not yet been clearly recognized whether the alteration is a cause or consequence of the disease. This calls for a thorough understanding of structure and mechanism of lncRNAs, their molecular interactions and development of novel quantitative assays to screen for drugs. Nonetheless, lncRNAs offer new hope for novel treatment options and in the near future it is expected that many of the lncRNAs may end up as strong diagnostic tools for cancer detection and patient management in the clinic. Because of the increasing number of cancer cases and its incurable nature, there is always a need for novel biomarkers for diagnosis, prognosis and therapy.

Key oncogenic and tumor suppressor lncRNAs suggested as potential biomarkers/therapeutic targets are summarized in Table 1.

## Conclusions and future perspectives

Cancer, being an incurable disease so far, needs novel and effective biomarkers and therapeutic strategies. It is becoming increasingly apparent that deregulated lncRNAs form a new stratum of intricacy in the molecular makeup of human diseases. Their role and mode of action in various signaling pathways during normal and disease conditions is being dissected meticulously and their significance is being acknowledged widely. LncRNAs are strongly associated with clinico-pathological outcome and prognosis of various diseases, more particularly in cancers and furnishing fresh therapeutic possibilities. They are generally expressed in tissue specific manner and exhibit aberrant expression in cancers. Therefore, targeting and either down-regulating or up-regulating specific lncRNAs in malignancies may not have deleterious side effects on normal cells. Thus, of late, both academia and biotech companies are turning their attention towards these novel and possibly personalized treatment options and trying to develop biological/nucleic acid drugs [282].

Various companies/organizations like RaNa Therapeutics, CuRNA, Sarepta, Smart Therapeutics, Allen Institution for Brain Science, Regulus, Miragen Therapeutics, Santaris Pharma etc. are pioneering the ncRNA based medicines. Soon we may see the time when lncRNA signature becomes a routine diagnostic test for diverse diseases, followed up by RNA based therapy curing the hitherto incurable diseases like cancer.
